# *catena*-Poly[bis­(4-amino­pyridinium) [[tetra­aqua­nickel(II)]-μ-benzene-1,2,4,5-tetra­carboxyl­ato] dihydrate]

**DOI:** 10.1107/S1600536809034746

**Published:** 2009-09-12

**Authors:** Archimede Rotondo, Giuseppe Bruno, Fabio Messina, Francesco Nicoló

**Affiliations:** a Dipartimento di Chimica Inorganica, Chimica Analitica e Chimica Fisica, Universitá di Messina, Salita Sperone, 31-98166 Messina, Italy

## Abstract

The asymmetric unit of the title compound, {(C_5_H_7_N_2_)_2_[Ni(C_10_H_2_O_8_)(H_2_O)_4_]·2H_2_O}_*n*_, contains an Ni^II^ atom, two water mol­ecules of coordination, one half of a benzene-1,2,4,5-tetra­carboxyl­ate (btec) anionic ligand, one 4-amino­pyridinium cation (papy) and an uncoordinated water mol­ecule. The metal center lies on an inversion center and adopts an octa­hedral geometry with the carboxyl­ate groups tilted out of the mean plane formed by the btec. In the crystal, mol­ecules are linked into one-dimensional coordination polymers running along the *ac* diagonal. The crystal structure is consolidated by N—H⋯O and O—H⋯O hydrogen bonds.

## Related literature

For background to 1,2,4,5-benzene-tetra­carboxyl­ate, see: Du *et al.* (2007 ▶); Fang *et al.* (2008 ▶); Loiseau *et al.* (2005 ▶); Ruiz-Pérez *et al.* (2004 ▶); Stephenson & Hardie (2006 ▶); Wang *et al.* (2005 ▶). For related structures, see: Majumder *et al.* (2006 ▶).
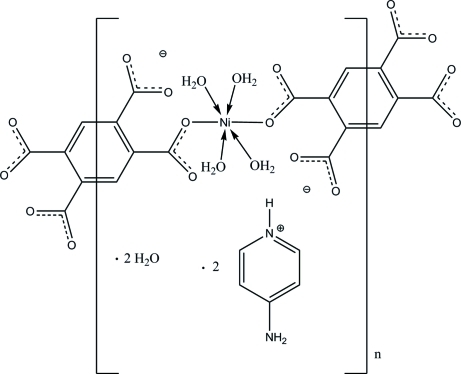


## Experimental

### 

#### Crystal data

(C_5_H_7_N_2_)_2_[Ni(C_10_H_2_O_8_)(H_2_O)_4_]·2H_2_O
*M* *_r_* = 607.17
Triclinic, 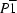

*a* = 7.2115 (1) Å
*b* = 9.3470 (1) Å
*c* = 10.6322 (2) Å
α = 112.720 (1)°
β = 108.830 (1)°
γ = 95.582 (1)°
*V* = 605.13 (2) Å^3^
*Z* = 1
Mo *K*α radiation
μ = 0.88 mm^−1^
*T* = 296 K
0.50 × 0.34 × 0.27 mm

#### Data collection

Bruker APEXII CCD diffractometer
Absorption correction: multi-scan (*SADABS*; Bruker, 2007 ▶) *T*_min_ = 0.729, *T*_max_ = 0.785
14068 measured reflections
2219 independent reflections
2203 reflections with *I* > 2σ(*I*)
*R*_int_ = 0.023

#### Refinement

*R*[*F* ^2^ > 2σ(*F* ^2^)] = 0.020
*wR*(*F* ^2^) = 0.055
*S* = 1.07
2219 reflections
187 parameters
H-atom parameters constrained
Δρ_max_ = 0.30 e Å^−3^
Δρ_min_ = −0.26 e Å^−3^

### 

Data collection: *APEX2* (Bruker, 2007 ▶); cell refinement: *SAINT* (Bruker, 2007 ▶); data reduction: *SAINT*; program(s) used to solve structure: *SHELXS97* (Sheldrick, 2008 ▶); program(s) used to refine structure: *SHELXL97* (Sheldrick, 2008 ▶); molecular graphics: *ORTEP-3 for Windows* (Farrugia, 1997 ▶) and *Mercury* (Macrae *et al.*, 2006 ▶); software used to prepare material for publication: *WinGX* (Farrugia, 1999 ▶) and *enCIFer* (Allen *et al.*, 2004 ▶).

## Supplementary Material

Crystal structure: contains datablocks global, I. DOI: 10.1107/S1600536809034746/pv2183sup1.cif

Structure factors: contains datablocks I. DOI: 10.1107/S1600536809034746/pv2183Isup2.hkl

Additional supplementary materials:  crystallographic information; 3D view; checkCIF report

## Figures and Tables

**Table 1:** Hydrogen-bond geometry (Å, °)

*D*—H⋯*A*	*D*—H	H⋯*A*	*D*⋯*A*	*D*—H⋯*A*
N6—H6⋯O1^i^	0.86	2.12	2.926 (1)	156
N12—H12*B*⋯O2	0.86	2.00	2.856 (2)	171
N12—H12*A*⋯O3*W*^ii^	0.86	2.19	3.048 (2)	174
O1*W*—H1*WA*⋯O2	0.85	1.81	2.634 (1)	163
O1*W*—H1*WB*⋯O3*W*^iii^	0.85	1.85	2.697 (1)	175
O2*W*—H2*WA*⋯O4^iv^	0.85	1.93	2.750 (1)	162
O2*W*—H2*WB*⋯O4^v^	0.85	1.90	2.732 (1)	165
O3*W*—H3*WA*⋯O3	0.85	1.86	2.694 (2)	168
O3*W*—H3*WB*⋯O3^vi^	0.85	2.12	2.911 (2)	154

Symmetry codes: (i) 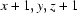; (ii) 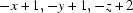; (iii) 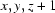; (iv) 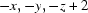; (v) 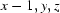; (vi) 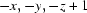.

**Fig. 1.**
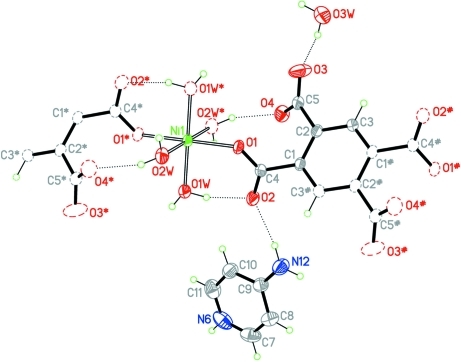
ORTEP view of I. Displacement ellipsoids are drawn at the 60% probability level. Symmetry codes for the dotted atoms: * -x, -y, -z + 2 and # -x + 1, -y + 1, -z + 2.

**Fig. 2.**
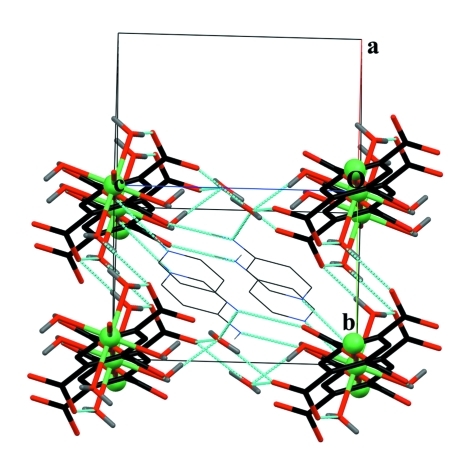
Molecular packing view of I along the diagonal of a and b axes. The crowded hydrogen bonding network is represented by dotted lines.

## supplementary crystallographic information

### Comment

The 1,2,4,5-benzene-tetracarboxylate (btec) is assessed to be a very versatile ligand able to achieve several coordination modes with different degrees of deprotonation (Du *et al.* 2007). The specific features of btec prompted researchers to explore different possible coordination polymers bearing specific features (Loiseau *et al.* 2005; Ruiz-Pérez *et al.* 2004), expecially in the presence of amines acting as ligands (Stephenson & Hardie, 2006; Wang *et al.* 2005) and as templates (Fang *et al.* 2008). Our attempts confirmed that the combination under standard conditins around pH 5 of btec, amines and metals, strictly depended on metals but also on the ancillary amines (yet to be published). The combination of equimolar solutions of nickel chloride, *p*-aminopyridine and di sodium dihydrogen btec under standard conditions gave two different kinds of green crystals, the rhomboidal-shaped ones (still under study) and the block-shaped ones which were found to be made up of the title compound, (I). The structure of (I) is isomorphous with the already described Co and Cu analogous (Majumder *et al.*, 2006).

The asymmetric unit of (I) is made by half of the metal centre with two coordinated water molecules and half btec ligand, one *p*-amino pyridinium cation (papy) and another uncoordinated water molecule. The metal centers present the octahedral coordination geometry, whereas carboxylate moieties are tilted out of the btec mean plane in order to develop specific inter-strand and intrastrand hydrogen bonding interactions. The crystallographic symmmetry results in monodimensional coordination polymers running along the diagonal of a and b crystallographic axes. These polymers are kept together by arrays of hydrogen bound papy cations (Fig. 2). The overal paking is obviously stabilized by a crowded hydrogen bonding network (Table 1).

### Experimental

An aqueous solution of disodium-dihydrogen 1,2,4,5-benzene tetracarboxylate (25 mmol) was slowly added to an equimolar aqueous solution of NiCl_2_ (50 mmol). Then an equimolar aqueous solution of *p*-aminopyridine was added to the mixture. The clear green solution at pH = 5.15 was left covered. After few days a white solid was separed from two different kind of green crystals. The rhomboidal-shaped crystals are under study (Bruno & Rotondo, to be pubblished), whereas the block-shaped ones were identified as I.

### Refinement

All hydrogen atoms were located in the difference map and were included in the refinements at geometrically idealized positions in the 'riding mode' with distances O–H, N–H and C–H fixed at 0.85, 0.86 and 0.93 Å, respectively, with temperature factors *U*_iso_(H) = 1.2 *U*_eq_(C/N) and 1.5 *U*_eq_(O).

### Crystal data

**Table:**

(C_5_H_7_N_2_)_2_[Ni(C_10_H_2_O_8_)(H_2_O)_4_]·2H_2_O	*Z* = 1
*M**_r_* = 607.17	*F*(000) = 316
Triclinic, *P*1	*D*_x_ = 1.666 Mg m^−^^3^
Hall symbol: -P 1	Mo *K*α radiation, λ = 0.71073 Å
*a* = 7.2115 (1) Å	Cell parameters from 9901 reflections
*b* = 9.3470 (1) Å	θ = 2.4–31.7°
*c* = 10.6322 (2) Å	µ = 0.88 mm^−^^1^
α = 112.720 (1)°	*T* = 296 K
β = 108.830 (1)°	Block, green
γ = 95.582 (1)°	0.5 × 0.34 × 0.27 mm
*V* = 605.13 (2) Å^3^	

### Data collection

**Table:**

Bruker APEXII CCD diffractometer	2219 independent reflections
graphite	2203 reflections with *I* > 2σ(*I*)
Detector resolution: 9 pixels mm^-1^	*R*_int_ = 0.023
φ and ω scans	θ_max_ = 25.4°, θ_min_ = 3.0°
Absorption correction: multi-scan (*SADABS*; Bruker, 2007)	*h* = −8→8
*T*_min_ = 0.729, *T*_max_ = 0.785	*k* = −11→11
14068 measured reflections	*l* = −12→12

### Refinement

**Table:**

Refinement on *F*^2^	Primary atom site location: structure-invariant direct methods
Least-squares matrix: full	Secondary atom site location: difference Fourier map
*R*[*F*^2^ > 2σ(*F*^2^)] = 0.020	Hydrogen site location: inferred from neighbouring sites
*wR*(*F*^2^) = 0.055	H-atom parameters constrained
*S* = 1.07	*w* = 1/[σ^2^(*F*_o_^2^) + (0.0286*P*)^2^ + 0.2508*P*] where *P* = (*F*_o_^2^ + 2*F*_c_^2^)/3
2219 reflections	(Δ/σ)_max_ = 0.001
187 parameters	Δρ_max_ = 0.3 e Å^−^^3^
0 restraints	Δρ_min_ = −0.26 e Å^−^^3^

### Special details

**Table:**

Geometry. All e.s.d.'s (except the e.s.d. in the dihedral angle between two l.s. planes) are estimated using the full covariance matrix. The cell e.s.d.'s are taken into account individually in the estimation of e.s.d.'s in distances, angles and torsion angles; correlations between e.s.d.'s in cell parameters are only used when they are defined by crystal symmetry. An approximate (isotropic) treatment of cell e.s.d.'s is used for estimating e.s.d.'s involving l.s. planes.
Refinement. Refinement of *F*^2^ against ALL reflections. The weighted *R*-factor *wR* and goodness of fit *S* are based on *F*^2^, conventional *R*-factors *R* are based on *F*, with *F* set to zero for negative *F*^2^. The threshold expression of *F*^2^ > σ(*F*^2^) is used only for calculating *R*-factors(gt) *etc*. and is not relevant to the choice of reflections for refinement. *R*-factors based on *F*^2^ are statistically about twice as large as those based on *F*, and *R*- factors based on ALL data will be even larger.

### Fractional atomic coordinates and isotropic or equivalent isotropic displacement parameters (Å^2^)

**Table:**

	*x*	*y*	*z*	*U*_iso_*/*U*_eq_	
Ni1	0	0	1	0.01722 (8)	
C1	0.42401 (18)	0.39111 (14)	1.04216 (14)	0.0178 (2)	
C2	0.43069 (18)	0.33541 (15)	0.90138 (14)	0.0180 (2)	
C3	0.50542 (18)	0.44605 (15)	0.86090 (14)	0.0188 (3)	
H3	0.5083	0.4106	0.7671	0.023*	
C4	0.32515 (19)	0.28168 (14)	1.08810 (14)	0.0186 (3)	
C5	0.36527 (19)	0.15800 (15)	0.79578 (14)	0.0210 (3)	
O1	0.14545 (13)	0.20052 (10)	1.00054 (10)	0.02033 (19)	
O2	0.42075 (15)	0.28245 (12)	1.20874 (11)	0.0319 (2)	
O3	0.2586 (2)	0.11600 (13)	0.66391 (12)	0.0458 (3)	
O4	0.42488 (15)	0.06463 (11)	0.84885 (11)	0.0282 (2)	
N6	0.9034 (2)	0.28646 (18)	1.77371 (15)	0.0397 (3)	
H6	0.9397	0.244	1.8336	0.048*	
C7	0.9976 (2)	0.4382 (2)	1.81496 (17)	0.0379 (4)	
H7	1.1004	0.4962	1.9087	0.046*	
C8	0.9467 (2)	0.50850 (18)	1.72376 (16)	0.0318 (3)	
H8	1.0126	0.6143	1.7554	0.038*	
C9	0.7933 (2)	0.42135 (17)	1.58003 (15)	0.0263 (3)	
C10	0.6936 (2)	0.26379 (18)	1.54195 (17)	0.0323 (3)	
H10	0.5874	0.2034	1.4502	0.039*	
C11	0.7530 (3)	0.2008 (2)	1.6396 (2)	0.0385 (4)	
H11	0.6883	0.0963	1.6131	0.046*	
N12	0.7487 (2)	0.48454 (16)	1.48525 (14)	0.0366 (3)	
H12A	0.8135	0.5801	1.5109	0.044*	
H12B	0.6549	0.43	1.3981	0.044*	
O1W	0.18379 (14)	0.05465 (11)	1.21508 (10)	0.0249 (2)	
H1WA	0.2767	0.1294	1.2294	0.037*	
H1WB	0.1353	0.0931	1.2806	0.037*	
O2W	−0.21078 (14)	0.11312 (11)	1.06521 (11)	0.0250 (2)	
H2WA	−0.2557	0.0702	1.1104	0.038*	
H2WB	−0.3121	0.1088	0.9937	0.038*	
O3W	0.05029 (18)	0.17265 (13)	0.43489 (12)	0.0358 (3)	
H3WA	0.1248	0.1693	0.5134	0.054*	
H3WB	−0.0649	0.1083	0.4016	0.054*	

### Atomic displacement parameters (Å^2^)

**Table:**

	*U*^11^	*U*^22^	*U*^33^	*U*^12^	*U*^13^	*U*^23^
Ni1	0.01682 (13)	0.01646 (12)	0.01797 (13)	−0.00033 (9)	0.00487 (9)	0.00990 (9)
C1	0.0150 (6)	0.0182 (6)	0.0194 (6)	0.0003 (5)	0.0045 (5)	0.0102 (5)
C2	0.0159 (6)	0.0171 (6)	0.0190 (6)	0.0011 (5)	0.0046 (5)	0.0085 (5)
C3	0.0188 (6)	0.0199 (6)	0.0172 (6)	0.0013 (5)	0.0067 (5)	0.0088 (5)
C4	0.0212 (6)	0.0155 (6)	0.0190 (6)	0.0018 (5)	0.0079 (5)	0.0082 (5)
C5	0.0220 (6)	0.0179 (6)	0.0217 (6)	0.0010 (5)	0.0090 (5)	0.0079 (5)
O1	0.0182 (4)	0.0195 (4)	0.0217 (4)	−0.0017 (3)	0.0049 (4)	0.0115 (4)
O2	0.0300 (5)	0.0348 (6)	0.0243 (5)	−0.0091 (4)	−0.0002 (4)	0.0193 (4)
O3	0.0665 (8)	0.0240 (5)	0.0223 (5)	0.0006 (5)	−0.0037 (5)	0.0059 (4)
O4	0.0328 (5)	0.0210 (5)	0.0337 (5)	0.0077 (4)	0.0129 (4)	0.0150 (4)
N6	0.0492 (8)	0.0541 (9)	0.0376 (8)	0.0307 (7)	0.0234 (7)	0.0323 (7)
C7	0.0351 (8)	0.0506 (10)	0.0244 (7)	0.0195 (7)	0.0084 (6)	0.0136 (7)
C8	0.0294 (8)	0.0299 (7)	0.0264 (7)	0.0065 (6)	0.0061 (6)	0.0073 (6)
C9	0.0253 (7)	0.0269 (7)	0.0251 (7)	0.0072 (6)	0.0084 (6)	0.0111 (6)
C10	0.0324 (8)	0.0284 (7)	0.0303 (8)	0.0030 (6)	0.0082 (6)	0.0120 (6)
C11	0.0470 (10)	0.0332 (8)	0.0481 (10)	0.0153 (7)	0.0256 (8)	0.0239 (7)
N12	0.0378 (7)	0.0321 (7)	0.0301 (7)	−0.0024 (6)	−0.0001 (6)	0.0176 (6)
O1W	0.0239 (5)	0.0274 (5)	0.0216 (5)	−0.0017 (4)	0.0062 (4)	0.0132 (4)
O2W	0.0231 (5)	0.0244 (5)	0.0301 (5)	0.0040 (4)	0.0106 (4)	0.0149 (4)
O3W	0.0413 (6)	0.0364 (6)	0.0328 (6)	0.0062 (5)	0.0154 (5)	0.0185 (5)

### Geometric parameters (Å, °)

**Table:**

Ni1—O1	2.054 (1)	N6—H6	0.86
Ni1—O1^i^	2.054 (1)	C7—C8	1.347 (2)
Ni1—O1W	2.063 (1)	C7—H7	0.93
Ni1—O1W^i^	2.063 (1)	C8—C9	1.412 (2)
Ni1—O2W^i^	2.082 (1)	C8—H8	0.93
Ni1—O2W	2.082 (1)	C9—N12	1.3249 (19)
C1—C3^ii^	1.3917 (18)	C9—C10	1.413 (2)
C1—C2	1.4012 (18)	C10—C11	1.357 (2)
C1—C4	1.5040 (16)	C10—H10	0.93
C2—C3	1.3894 (17)	C11—H11	0.93
C2—C5	1.5168 (17)	N12—H12A	0.86
C3—C1^ii^	1.3917 (18)	N12—H12B	0.86
C3—H3	0.93	O1W—H1WA	0.8491
C4—O2	1.2416 (16)	O1W—H1WB	0.8517
C4—O1	1.2673 (16)	O2W—H2WA	0.8491
C5—O3	1.2376 (17)	O2W—H2WB	0.8517
C5—O4	1.2496 (17)	O3W—H3WA	0.8491
N6—C11	1.342 (2)	O3W—H3WB	0.8517
N6—C7	1.344 (2)		
			
O1—Ni1—O1^i^	180	C4—O1—Ni1	124.64 (8)
O1—Ni1—O1W	94.96 (4)	C11—N6—C7	120.39 (14)
O1^i^—Ni1—O1W	85.04 (4)	C11—N6—H6	119.8
O1—Ni1—O1W^i^	85.04 (4)	C7—N6—H6	119.8
O1^i^—Ni1—O1W^i^	94.96 (4)	N6—C7—C8	121.61 (15)
O1W—Ni1—O1W^i^	180	N6—C7—H7	119.2
O1—Ni1—O2W^i^	87.65 (4)	C8—C7—H7	119.2
O1^i^—Ni1—O2W^i^	92.35 (4)	C7—C8—C9	119.94 (15)
O1W—Ni1—O2W^i^	87.40 (4)	C7—C8—H8	120
O1W^i^—Ni1—O2W^i^	92.60 (4)	C9—C8—H8	120
O1—Ni1—O2W	92.35 (4)	N12—C9—C8	121.08 (14)
O1^i^—Ni1—O2W	87.65 (4)	N12—C9—C10	122.01 (13)
O1W—Ni1—O2W	92.60 (4)	C8—C9—C10	116.90 (13)
O1W^i^—Ni1—O2W	87.40 (4)	C11—C10—C9	119.85 (15)
O2W^i^—Ni1—O2W	180	C11—C10—H10	120.1
C3^ii^—C1—C2	120.00 (11)	C9—C10—H10	120.1
C3^ii^—C1—C4	118.01 (11)	N6—C11—C10	121.24 (15)
C2—C1—C4	121.77 (11)	N6—C11—H11	119.4
C3—C2—C1	118.62 (11)	C10—C11—H11	119.4
C3—C2—C5	119.85 (11)	C9—N12—H12A	120
C1—C2—C5	121.49 (11)	C9—N12—H12B	120
C2—C3—C1^ii^	121.37 (12)	H12A—N12—H12B	120
C2—C3—H3	119.3	Ni1—O1W—H1WA	98.6
C1^ii^—C3—H3	119.3	Ni1—O1W—H1WB	116.7
O2—C4—O1	125.75 (11)	H1WA—O1W—H1WB	107.6
O2—C4—C1	118.41 (11)	Ni1—O2W—H2WA	109.6
O1—C4—C1	115.78 (11)	Ni1—O2W—H2WB	114
O3—C5—O4	124.70 (12)	H2WA—O2W—H2WB	107.6
O3—C5—C2	118.15 (12)	H3WA—O3W—H3WB	107.6
O4—C5—C2	117.15 (11)		
			
C3^ii^—C1—C2—C3	−1.1 (2)	O2—C4—O1—Ni1	−20.66 (18)
C4—C1—C2—C3	173.38 (11)	C1—C4—O1—Ni1	162.24 (8)
C3^ii^—C1—C2—C5	176.81 (11)	O1W—Ni1—O1—C4	21.65 (10)
C4—C1—C2—C5	−8.73 (18)	O1W^i^—Ni1—O1—C4	−158.35 (10)
C1—C2—C3—C1^ii^	1.1 (2)	O2W^i^—Ni1—O1—C4	−65.53 (10)
C5—C2—C3—C1^ii^	−176.83 (11)	O2W—Ni1—O1—C4	114.47 (10)
C3^ii^—C1—C4—O2	−54.95 (17)	C11—N6—C7—C8	−0.7 (2)
C2—C1—C4—O2	130.48 (13)	N6—C7—C8—C9	−1.1 (2)
C3^ii^—C1—C4—O1	122.37 (13)	C7—C8—C9—N12	−176.02 (14)
C2—C1—C4—O1	−52.20 (16)	C7—C8—C9—C10	2.8 (2)
C3—C2—C5—O3	−45.83 (18)	N12—C9—C10—C11	175.97 (15)
C1—C2—C5—O3	136.30 (14)	C8—C9—C10—C11	−2.9 (2)
C3—C2—C5—O4	133.70 (13)	C7—N6—C11—C10	0.7 (2)
C1—C2—C5—O4	−44.16 (17)	C9—C10—C11—N6	1.2 (2)

Symmetry codes: (i) −*x*, −*y*, −*z*+2; (ii) −*x*+1, −*y*+1, −*z*+2.

### Hydrogen-bond geometry (Å, °)

**Table:**

*D*—H···*A*	*D*—H	H···*A*	*D*···*A*	*D*—H···*A*
N6—H6···O1^iii^	0.86	2.12	2.926 (1)	156
N12—H12B···O2	0.86	2.00	2.856 (2)	171
N12—H12A···O3W^ii^	0.86	2.19	3.048 (2)	174
O1W—H1WA···O2	0.85	1.81	2.634 (1)	163
O1W—H1WB···O3W^iv^	0.85	1.85	2.697 (1)	175
O2W—H2WA···O4^i^	0.85	1.93	2.750 (1)	162
O2W—H2WB···O4^v^	0.85	1.90	2.732 (1)	165
O3W—H3WA···O3	0.85	1.86	2.694 (2)	168
O3W—H3WB···O3^vi^	0.85	2.12	2.911 (2)	154

Symmetry codes: (iii) *x*+1, *y*, *z*+1; (ii) −*x*+1, −*y*+1, −*z*+2; (iv) *x*, *y*, *z*+1; (i) −*x*, −*y*, −*z*+2; (v) *x*−1, *y*, *z*; (vi) −*x*, −*y*, −*z*+1.
